# Gravity Anomaly Characteristics and Tectonic Implications of the Tangshan Seismic Zone

**DOI:** 10.3390/s26134113

**Published:** 2026-06-29

**Authors:** Minghui Zhang, Jiapei Wang, Guiju Wu, Hongbo Tan, Li Zhang

**Affiliations:** 1 Key Laboratory of Earthquake Geodesy, Institute of Seismology, China Earthquake Administration, Wuhan 430071, China; zhangminghui@hubdzj.gov.cn (M.Z.); wuguiju@hubdzj.gov.cn (G.W.); tanhongbo@hubdzj.gov.cn (H.T.); zhanglly0919@163.com (L.Z.); 2 Gravitation and Earth Tide, National Observation and Research Station, Wuhan 430071, China

**Keywords:** Tangshan earthquake, Bouguer gravity anomaly, multi-scale wavelet decomposition, deep and shallow structures

## Abstract

A catastrophic Ms7.8 earthquake occurred in Tangshan in 1976 at a focal depth of approximately 12 km, resulting in severe casualties and substantial economic losses. Given its unique tectonic setting, the seismogenic structure and dynamic genesis of the Tangshan earthquake have long remained a key research topic in seismotectonic studies. To better characterize the tectonic framework, seismogenic mechanisms, and deep–shallow dynamical coupling within the Tangshan seismic zone, we employ multi-scale wavelet decomposition on high-resolution residual gravity anomalies to isolate crustal structure signals across different depth ranges. Integrating these structural signatures with the spatial distribution of seismicity yields a comprehensive framework for interpreting the regional tectonic evolution. The Tangshan seismic zone is positioned within the intricate structural architecture of the Tangshan rhombic fault block, a system embedded within the broader context of the North China Craton (NCC) destruction. Seismicity displays a distinct preferred orientation, with events concentrated along block-bounding faults and gravity anomaly gradient zones. With increasing wavelet decomposition levels, the gravity anomalies exhibit a systematic transition from spatially dispersed patterns associated with shallow structures to more concentrated features reflecting deeper geological domains. Shallow anomalies from the first to third decomposition orders, which are primarily controlled by Quaternary sedimentary layers, show a fragmented distribution that corresponds well with the development of local flower structures and the occurrence of diffuse shallow seismicity. The fourth- to seventh-order anomalies clearly delineate the rhombic block and its bounding peripheral faults, highlighting the structural intersections that hosted the Tangshan mainshock and its associated aftershock sequence. In contrast, the eighth- to tenth-order deep-seated anomalies corresponding to deeper structural levels exhibit pronounced coalescence, effectively imaging mantle upwelling and large-scale density heterogeneities within the lithospheric mantle. These concentrated gravity highs are closely coupled with mantle thermal activity, whose upward ascent induces thermal weakening of the lower crust and facilitates progressive stress transfer toward shallower crustal levels. Concurrently, frictional locking of shallow high-angle faults promotes intense stress accumulation within the rigid basement. The interplay between deep-seated dynamic concentration and shallow structural confinement ultimately triggers the catastrophic coseismic rupture responsible for the Tangshan earthquake. By delineating the structural transition from deep-seated aggregation centers to shallow dispersed fracture zones, this study establishes a robust framework for assessing seismogenic environments and regional seismic hazard potential across the progressively destroyed NCC.

## 1. Introduction

As a global paradigm for craton destruction, the NCC has long stood as a focal point in geoscientific research, with particular emphasis on its deep dynamical processes and the seismogenic mechanisms underlying major earthquakes. The Tangshan seismic zone is situated in the northeastern part of the NCC, at the junction of the Yanshan Uplift and the Bohai Bay Basin. Characterized by its complex deep structures and intense tectonic activity. This region serves as a geodynamic paradigm for elucidating the regulatory role of regional geodynamics within the broader framework of cratonic destruction [1].

From the regional scale of the NCC to the localized Tangshan source area, multidisciplinary geophysical investigations have elucidated the lithospheric architecture and geodynamic framework. Magnetotelluric (MT) studies indicate that the interaction between asthenospheric upwelling beneath the Bohai Bay Basin and the rigid blocks of the Yanshan Belt constitutes the primary deep driving force for major seismicity in central and eastern China [2,3]. Investigations into the electrical structure of the central NCC further reveal significant low-resistivity anomalies within the northern lithosphere; these high-conductivity features reflect thermal upwelling and fluid activity associated with cratonic destruction, thereby providing critical electrical evidence for lithospheric thinning and transformation [4]. Specifically, a prominent high-conductivity body identified beneath the Tangshan source area suggests that localized thermal upwelling triggered by the subduction of the Western Pacific Plate serves as a key dynamic factor in the initiation of the Tangshan earthquake [5]. With respect to seismic sounding and tomography, the crustal structural framework of central–eastern China has been established to clarify the deep tectonic origins of large earthquakes, underscoring the control that cratonic destruction exerts on regional seismicity [6,7]. Through the joint inversion of multi-source geophysical data, researchers have refined both the evolutionary extent and geodynamic mechanisms of lithospheric destruction in the NCC, providing integrated constraints on crust–mantle interactions [8]. Furthermore, the application of machine learning for high-precision seismic phase picking has facilitated high-resolution tomographic imaging of the uppermost mantle velocity structure, yielding new evidence for mantle migration and interior dynamic evolution [9]. Long-term shear-wave splitting analyses have also revealed complex seismic anisotropy beneath the NCC, which clarifies the relationship between mantle flow fields and lithospheric deformation modes [10]. Complementary studies employing velocity and attenuation tomography have delineated crust–mantle heterogeneities and lithospheric modification, thereby further substantiating the processes of cratonic destruction and transformation [8,11].

Focusing on high-resolution detection within the Tangshan source area, research efforts have prioritized the characterization of medium properties and their regulatory control over seismic distribution. Previous deep seismic reflection and refraction profiles identified significant velocity anomalies in the middle and upper crust, which clarified the deep extension of fault zones and the nature of medium heterogeneity [12,13]. More recently, ambient noise tomography has further refined the 3D velocity structure of the Tangshan fault zone, revealing that seismicity tends to cluster along the margins of high-resistivity and high-velocity bodies [14]. Furthermore, integrated geochemical and geophysical analyses have elucidated material migration patterns during tectonic activities, confirming that deep thermal upwelling and crust–mantle heterogeneity play a decisive role in shaping the regional seismogenic environment [15]. The shallow morphology of seismogenic structures, fault locking, and the spatio-temporal evolution of earthquakes remain focal points. Geological surveys and shallow exploration have refined the non-uniform distribution and displacement of surface rupture zones [16], confirming that the seismogenic fault exhibits right-lateral strike–slip characteristics with a thrust component, along with typical flower structures in the shallow crust [17,18]. The coupling between deep stress accumulation, locked fault segments, and shallow surface ruptures has also been well established [19,20]. In seismological studies, previous investigations delineated the propagation of the main rupture and the distribution of focal depths [21,22]. Recently, high-precision relocation techniques have meticulously identified the 3D geometric segmentation and conjugate branching of the fault zone [23,24]. Investigations into the Guye earthquake further reveal complex interactions between the seismogenic fault and secondary fractures [25]. Stress evolution analyses suggest that the aftershocks of the Tangshan earthquake may persist for 65–100 years, with this persistence correlating with areas of increased Coulomb failure stress [26]. Additionally, geoelectrical resistivity (GER) anomaly observations confirm the control of deep fluid and stress migration over seismic precursors [27].

Spatially, the seismicity exhibits pronounced heterogeneity. The vast majority of events—including the locus of the 1976 Ms7.8 Tangshan earthquake—are densely clustered along the northern margin of the North China Basin. This concentration highlights the intense tectonic activity of buried structures beneath thick sedimentary cover. In contrast, seismic frequency decreases markedly within the northern Yanshan Uplift. The sharp lithological and rheological contrasts between the ancient, rigid crystalline basement to the north and the deep Cenozoic sedimentary basin to the south create significant lateral crustal heterogeneity, facilitating stress localization at the basin–range junction. Furthermore, persistent seismic activity at the land–sea transition (southern coastal Ninghe and Tanghai) suggests that this seismic belt is driven by a deep-seated, large-scale regional tectonic stress field. The Tangshan fault zone is a major active fault within the study area. It trends generally NE and consists of multiple subparallel fault strands that collectively form a fault zone architecture. This fault zone not only governs the topographic and geomorphic configuration of the Tangshan region but is also considered the primary seismogenic structure responsible for the 1976 Tangshan Ms7.8 earthquake.

The study area is characterized by an intricate network of faults, with the Tangshan fault and its peripheral fractures serving as the primary seismogenic and controlling structures. Seismicity exhibits a pronounced NE-trending linear distribution, showing strong spatial alignment with the orientation of the Tangshan fault zone. The most intense seismic clustering occurs within the Tangshan–Guye–Luanxian region, which notably coincides with the Cenozoic sedimentary depocenter and the intersections of multiple variably oriented fault systems. This spatial overlap underscores the role of these complex tectonic nodes as focal points for stress concentration and subsequent energy release.

Gravity exploration—characterized by its operational simplicity, cost-effectiveness, broad spatial coverage, and high efficiency—remains a fundamental method for investigating regional tectonics. Previous gravity studies pioneered the investigation of the 3D lithospheric density distribution in North China, laying the groundwork for a preliminary structural framework [28]. Building on this foundation, subsequent research has focused on the 3D density structure of the NCC lithosphere, elucidating spatial variations in density heterogeneity across the craton [29]. For the Beijing–Tianjin–Hebei metropolitan region, refined crustal structures derived from gravity field inversion have clarified layered density variations, providing critical data to advance understanding of deep tectonic evolution [30]. With respect to the Sanhe–Pinggu Ms8.0 seismic zone, 3D density inversion has enabled the precise identification of density anomalies, revealing an inherent correlation between density structure and tectonic activity [31]. Furthermore, within the Beijing Plain, gravity anomalies have been effectively utilized for fault identification, establishing a clear spatial correspondence between fault traces and density anomaly zones [32].

While these geophysical studies have established a foundational understanding of the Tangshan seismic zone, several critical gaps remain in elucidating the underlying seismogenic mechanisms. Despite multidisciplinary evidence confirming deep dynamical loading, the lateral heterogeneity of deep–shallow structural coupling remains a subject of debate, which hinders the quantitative characterization of interactions between tectonic units. Furthermore, constrained by station density, MT sounding and seismic tomography possess limited lateral resolution within the source area, impeding the refined investigation of medium heterogeneity. Conventional gravity studies have predominantly focused on constructing 3D density structures but lack depth-resolved multi-scale decomposition of gravity anomalies. The inherent superposition of multi-source anomaly signals further introduces significant uncertainties into density modeling. Wavelet decomposition has played a significant role in gravimetry, allowing for the separation of various components of the gravity field [33,34]. This technique has been extensively employed in a wide range of applications, including tectonic activity analysis and gravity inversion [1,35].

Building on this research background, the present study utilizes high-precision, high-resolution Bouguer gravity anomalies (BGAs) data from the Tangshan seismic zone and its adjacent regions. By employing multi-scale wavelet decomposition, multi-level potential field separation of the gravity field is conducted to effectively isolate anomaly signals originating from different source depths. Through the analysis of the spatial evolution of gravity anomalies across different scales, the coupling relationships between deep-seated anomalies, the distribution of the Tangshan rhombic fault block, mantle thermal migration, and the spatial patterns of seismic sequences are investigated. This work aims to provide more refined geophysical insights for identifying seismogenic environments within the craton destruction region of NCC.

## 2. Materials and Methods

### 2.1. Geological Setting and Seismicity

As one of the world’s oldest continental nuclei, the NCC has experienced extensive lithospheric modification and destruction since the Mesozoic Era. Its eastern domain, which includes the Bohai Bay Basin, the Yanshan Tectonic Belt, and the Zhangjiakou–Bohai Seismic Belt, features vigorous tectonic activity and frequent major earthquakes. This renders it a key natural laboratory for exploring cratonic destruction dynamics, deep geodynamic processes, and regional seismogenic conditions [2,3]. Tectonic evolution across this area is driven by complex interactions between deep geodynamic processes, such as Pacific Plate subduction and rollback, lithospheric delamination, and asthenospheric upwelling. Combined, these processes have shaped highly heterogeneous crust–mantle architecture and stress field distribution [3,11]. The 1976 Ms7.8 Tangshan earthquake represents the most devastating intraplate seismic disaster recorded in this region. Tectonically, the Tangshan seismic zone lies on the northern margin of the NCC, at the transitional boundary between the Yanshan Uplift and the North China Basin. It is also located east of the central segment of the great gravity gradient zone in eastern China [1]. Detailed research on local seismogenic structures and tectonic settings is critical for deciphering intraplate earthquake genesis, while also supplying fundamental support for regional seismic risk evaluation.

**Figure 1:**
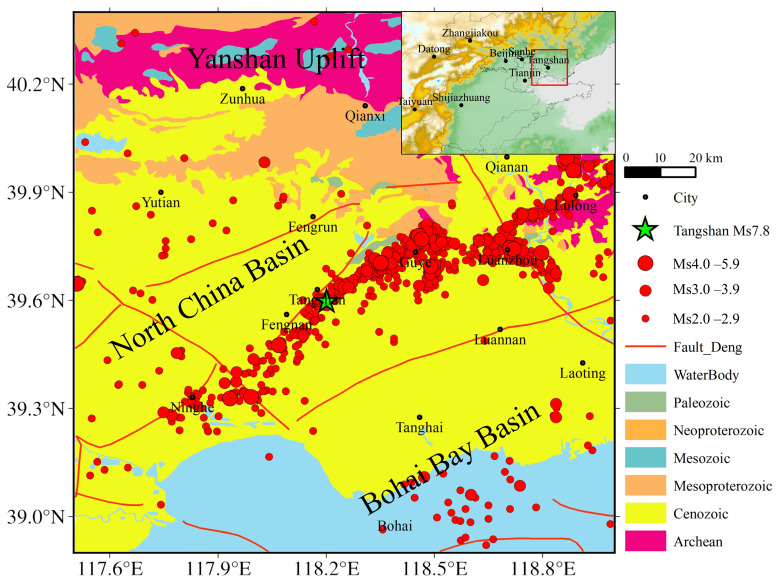
Geological setting and seismicity of the Tangshan region. The red box denotes the study area.

Figure 1 illustrates the regional geological setting and seismicity distribution of the Tangshan seismic zone and its adjacent regions. From a geological evolutionary perspective, the study area shows obvious north–south structural differences. The northern segment—including Zunhua, Qianxi, Qian’an, and Lulong—sits along the southern margin of the Yanshan Uplift, where the Archean crystalline basement is extensively exposed. Sporadic outcrops of Mesoproterozoic, Neoproterozoic, and Paleozoic to Mesozoic strata occur across this region. The inherent rigidity of this basement, combined with its considerable crustal thickness, forms a stable rigid tectonic block within the regional geodynamic system. In contrast, the south-central segment—encompassing Tangshan, Fengnan, Luannan, Tanghai, and the Bohai coastal area—is located at the northern terminus of the North China Basin. Under the influence of large-scale Cenozoic subsidence, the surface is overlain by a thick sequence of unconsolidated Cenozoic sediments, resulting in a structural environment where the basement is predominantly concealed.

### 2.2. Data Sources and Preprocessing

**Figure 2:**
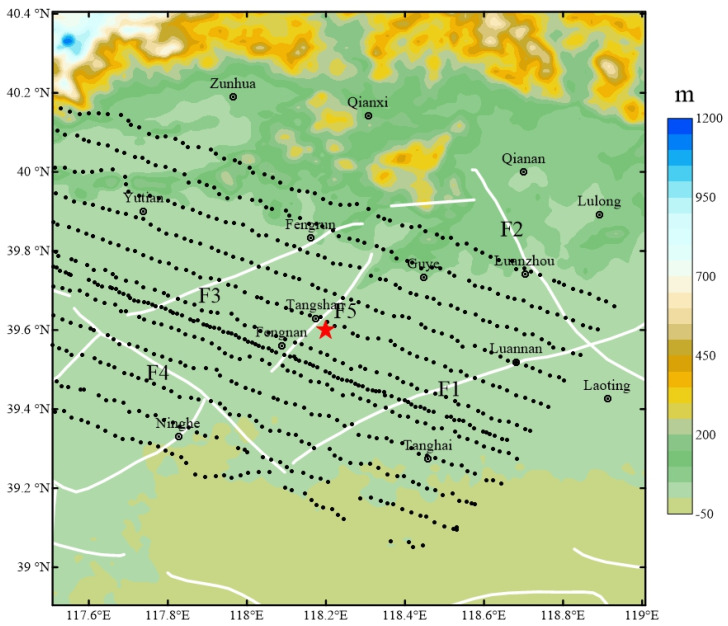
Topography, active faults and distribution of gravity measurement points in the Tangshan seismic zone and its adjacent areas. The background color represents the topographic elevation; White solid lines denote major active faults; black dots represent the distribution of mobile gravity stations; the red star indicates the epicenter of the 1976 Ms7.8 Tangshan earthquake. F1: Ninghe–Changli fault; F2: Luanzhou–Laoting fault; F3: Fengtai–Yejituo fault; F4: Jiyunhe fault; F5: Tangshan fault.

To investigate the gravity anomaly characteristics of the Tangshan seismic zone, we conducted a joint gravity and GNSS survey within the source area and its surrounding regions. A total of 733 field measurement points were established, with a spatial distribution illustrated in Figure 2. The survey was designed with a station spacing of approximately 1–2 km and a line spacing of roughly 5 km. The gravity measurement precision was better than 0.01 mGal, while GNSS positioning achieved horizontal and vertical accuracies better than 0.04 m and 0.06 m, respectively. The raw data underwent a rigorous processing sequence, including base station network adjustment, normal field correction, Bouguer reduction (incorporating Free-air and Bouguer plate corrections), and comprehensive terrain corrections (covering near, intermediate, and far zones). Following these reductions, the BGAs for each station were derived. To integrate these field measurements with the 1:200,000 regional BGAs map of East Hebei region (compiled by the Geophysical Survey Team of the Hebei Bureau of Geology and Mineral Resources in 1989), we performed coordinate transformations, systematic bias corrections, and least-squares adjustments within the overlapping regions. To eliminate potential edge artifacts at the data fusion interface, a variable-weight sliding smoothing method [35] was employed, ensuring spatial continuity and the reliability of the integrated gravity field.

This study presents a comprehensive seismic catalog for the Tangshan seismic zone (117.5° E~119° E, 38.9° N~40.4° N) covering the period from February 2008 to January 2026. The seismicity is dominated by small-to-moderate events, with earthquakes of 2.0 ≤ Ms ≤ 3.0 accounting for approximately 90% of the total; focal depths are primarily concentrated between 5 and 20 km. The historical earthquake data shown in the figure were obtained from the China Earthquake Networks Center, comprising a total of 1175 events. Historical seismic activity in the Tangshan earthquake region is primarily distributed within the Tangshan rhombic block bounded by deep-seated faults and near the positive density anomaly zones in the middle–upper crust. This region is located at the intersection of the Cangzhou sub-block boundary and the Zhangjiakou–Bohai Fault Zone. Strong earthquake activity in this area is jointly controlled by differential movements along active block boundaries and the upwelling of deep high-density materials.

The Ninghe–Changli Fault (F1) is a deep-seated fault trending NNE to NE. It is one of the four major boundary faults bounding the Tangshan rhombic block and constitutes an important tectonic boundary at the junction between the Yanshan fold belt and the North China rift basin. The fault exhibits strike–slip characteristics, with positive and negative density anomaly bodies (e.g., the Laoting Depression and the Luanzhou Uplift) distributed on both sides, reflecting the cutting effect of the fault. The Luanzhou–Laoting Fault (F2) trends NW and is likewise one of the four deep-seated boundary faults confining the Tangshan rhombic block (i.e., the Tangshan block depression). It cuts through the crust and, in terms of density structure, often serves as the boundary between different tectonic units (e.g., uplift and depression). The Fengtai–Yejituo Fault (F3) is a deep-seated fault trending NE and is also one of the four boundary faults of the Tangshan rhombic block (i.e., the Tangshan block depression). It plays a distinct role in delineating the density structure. The Jiyunhe Fault (F4) is an active fault trending NW and is also one of the four boundary faults bounding the Tangshan rhombic block. Its strike is generally consistent with the pre-earthquake gravity change gradient zone and divergence concentration zone. The Tangshan Fault (F5) is a complex fault zone with an NE trend, a high-angle westward dip, and thrust–strike–slip properties. It is the seismogenic structure of the 1976 Tangshan Ms7.8 earthquake. The fault penetrates the Moho in depth, exhibits a flower-like structure in the shallow part, and is influenced by magmatic intrusion at depth.

### 2.3. Characteristics of Bouguer Gravity Anomalies

**Figure 3:**
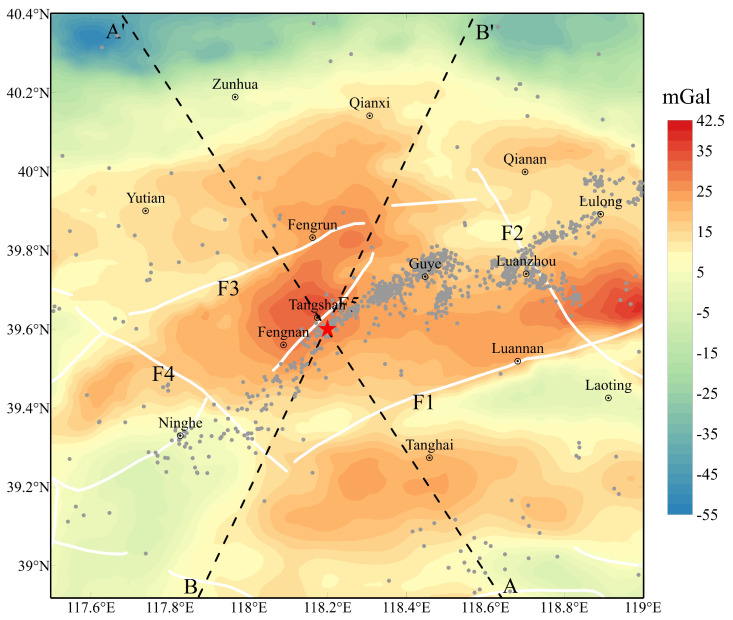
BGAs and seismicity distribution in the Tangshan seismic zone and its adjacent areas. Black dashed lines AA’ and BB’ represent extracted gravity profiles crossing the epicentral core and primary faults, the gray dots denote the historical seismicity data. The red star indicates the epicenter of the 1976 Tangshan Ms7.8 earthquake.

Figure 3 presents the refined BGAs map for the Tangshan seismic zone and adjacent areas (38.9° N~40.4° N, 117.5° E~119° E), based on data integration and mosaic processing. The BGAs exhibit a general decreasing trend from south to north. A prominent E-W trending BGAs gradient zone is developed along the northern Zunhua–Qian’an belt; the Yanshan Uplift to the north is characterized by negative anomalies, reflecting relative crustal thickening, whereas the North China Rift Basin to the south is dominated by positive anomalies. Notably, the epicentral area (Tangshan–Fengnan) exhibits a significant local gravity high (approximately 15~35 mGal), suggesting a basement uplift or a high-density lithogenic body at depth. In the southeastern sector near Laoting and Luannan, anomaly values fluctuate between −5~5 mGal, with localized highs northeast of Luannan. The western (Ninghe) and southern regions feature transitional values of approximately 15 mGal with relatively gentle contours. Integrated analysis of regional fault distribution and seismic spatial patterns reveals that seismicity is densely clustered along the NE-trending BGAs gradient zones between the Tangshan–Fengnan local gravity high and the surrounding lows. This seismic distribution aligns closely with the strike of the primary seismogenic fault (Tangshan Fault), underscoring that regions where deep-seated physical property contrasts intersect with active faults are highly susceptible to stress accumulation.

### 2.4. Principles of Multi-Scale Wavelet Decomposition

BGAs reflect the integrated response of superimposed density heterogeneities spanning various depths and scales. Traditional field separation methods often struggle to precisely decouple anomalies originating from distinct crustal levels. In contrast, multi-scale wavelet decomposition—leveraging its superior spatial and spectral localization—enables the layer-by-layer partitioning of gravity signals across different scale spaces. This approach facilitates the effective isolation of structural anomalies within shallow, intermediate, and deep crustal layers, providing robust potential-field constraints for refined tectonic interpretation.

Multi-scale wavelet decomposition effectively separates regional and residual gravity anomalies (RGAs) and extracts fine-scale detail anomalies. This process partitions the composite signal into components across multiple scales, where each component corresponds to the gravitational contribution of source bodies at specific depth ranges [36,37]. This relationship is mathematically expressed as

(1) $$\Delta g(x,y)=A_{N}(x,y)+\sum_{k=1}^{N}D_{k}(x,y)$$

where Δg(x,y) represents the BGA signal. $A_{N}(x,y)$ denotes the *N*-th order wavelet approximation component, reflecting deep-seated background anomalies at a regional scale. These anomalies correspond to lithospheric-scale tectonic features, such as Moho depth variations. $D_{k}(x,y)$ is the *k*-th order wavelet detail component, characterizing RGAs information; specifically, lower-order details correspond to density heterogeneities in shallow strata, while higher-order details reveal density variations extending from the mid-crust to the lower crust. The decomposition level *N*, is a manually selected parameter. According to the “Invariance Criterion of Lower-order Wavelet Details” in wavelet transforms, the extracted lower-order wavelet details remain consistent regardless of the choice of *N* [38]. The primary difference lies in the total number of wavelet detail components generated and the resulting *N*-th order approximation.

Establishing an accurate correspondence between wavelet decomposition components and the depth of subsurface structures is essential for deep tectonic interpretation. The burial depths of subsurface density anomalies can be inferred from the characteristics of their power spectrum in the frequency domain. By transforming the BGAs from the spatial to the frequency domain, utilizing the mathematical relationship between the physical attributes of the field source and its spectral decay to quantify depth. Specifically the radially averaged power spectrum of gravity anomalies exhibits a log-linear decay relationship [39], expressed as

(2) $$\ln P(k)=\ln P_{0}-4\pi hk$$

In the equation, *P*(*k*) represents the radially averaged power spectrum at wavenumber *k*, and *P*_0_ is the intercept term of the model, derived through linear regression fitting of the log-linear segments of the power spectrum. *h* denotes the equivalent average burial depth of the field source. The slope of the resulting fit is defined as −4*πh* indicating that the attenuation rate of the power spectrum is positively correlated with the burial depth of the field source.

## 3. Results

### 3.1. Transverse Multi-Scale Decomposition Characteristics

To better characterize the crustal architecture and the deep seismogenic drivers of the Tangshan Ms7.8 earthquake, the 2D multi-scale wavelet decomposition method is utilized to study the Tangshan seismic zone. The decomposition was implemented using the Biorthogonal wavelet basis due to its linear phase and compact support [40]. Utilizing the Mallat pyramidal algorithm, the method adheres to the “invariance criterion of lower-order details” [38]. This principle dictates that during an N-level discrete wavelet transform, each subsequent first-order decomposition isolates new higher-order details and adjusts the higher-order approximation without altering the previously established lower-order details. By sequentially repeating these first-order decompositions-extracting detail *D*_1_ and approximation *A*_1_, followed by the decomposition of *A*_1_ to yield *D*_2_ and *A*_2_, and repeating this process, we can successfully isolate anomalies generated by field sources at distinctly different depths.

**Figure 4:**
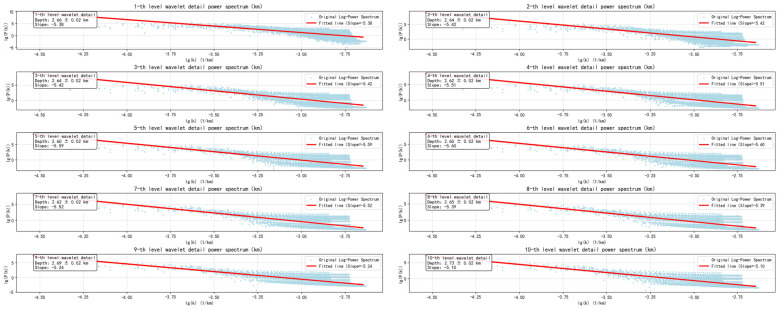
Power spectrum fitting and depth estimation plots for the wavelet detail components of the RGAs.

To further elucidate the physical significance of the wavelet details, power spectrum analysis was performed on the components obtained from the multi-scale decomposition. This analysis facilitates the determination of the approximate field source depths represented by each wavelet detail. Figure 4 displays the power spectrum fitting plots for the various wavelet orders. As indicated in the figure, the estimated source depths for the 1st through 10th order wavelet details are 2.66 km, 5.3 km, 7.94 km, 10.56 km, 13.16 km, 15.76 km, 18.38 km, 21.03 km, 23.72 km, and 26.45 km, respectively. The uncertainty of depth estimates in the study area is uniformly 0.02 km, obtained by calculating the standard deviation of the differences between the data points at each depth and the corresponding linear fitting curve.

**Figure 5:**
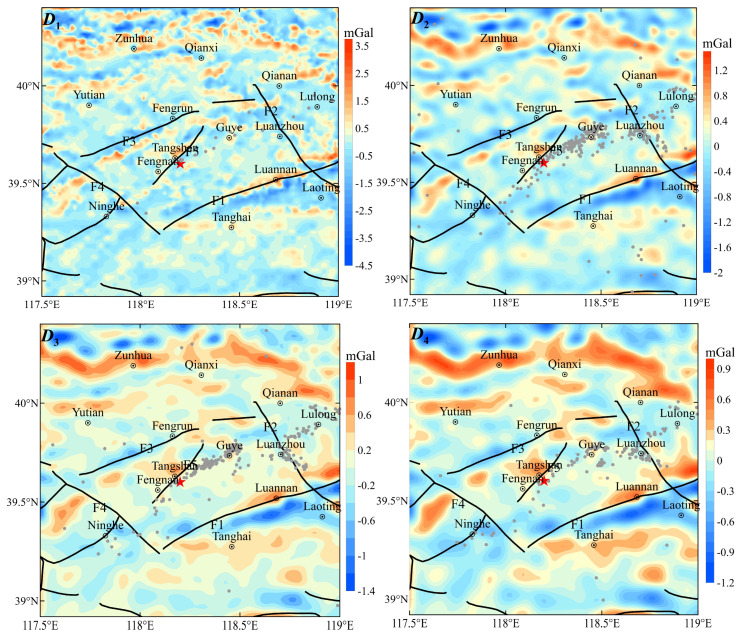
1st-to-4th order wavelet detail components (*D*_1_~*D*_4_) of the RGAs in the Tangshan seismic zone. The red star indicates the epicenter of the 1976 Tangshan Ms7.8 earthquake.

Figure 5 illustrates the 1st to 4th order wavelet detail components derived from the BGAs within the study area. The 1st and 2nd order wavelet details (*D*_1_ and *D*_2_) primarily reflect the distribution of shallow subsurface materials. The gravity field at these scales is characterized by high-frequency, fragmented, and alternating positive and negative anomalies that appear spatially incoherent, lacking prominent regional patterns. Negative anomalies are predominantly concentrated near the northern Yanshan Uplift and the Tanghai–Laoting area, correlating with localized depressions within the Yanshan Uplift and the Laoting Depression. These features primarily reflect Quaternary sedimentary thickness and the heterogeneity of near-surface structures in the seismic zone; notably, their correlation with seismic activity is relatively weak.

The 3rd and 4th order wavelet details (*D*_3_ and *D*_4_) primarily reflect the gravitational effects induced by the heterogeneous distribution of material density within the upper crust. In these components, the anomaly morphology transitions toward a banded distribution, with gradients between positive and negative gravity anomalies aligning closely with the traces of deep-seated large faults. This suggests that these RGAs gradient zones arise from density contrasts associated with crustal fragmentation during fault movements within the upper crust. Positive anomalies are predominantly concentrated in the northern Zunhua–Qianxi–Qian’an region, the vicinity of Fengnan–Tangshan–Guye–Luannan, and the southern Tanghai area; notably, the spatial extent of these positive anomalies expands with increasing depth. The Laoting–North Tanghai region is characterized by broad, subtle negative anomalies extending northwestward toward southern Fengnan, reflecting the regional configuration of sedimentary depressions. The Ninghe–Changli fault and Fengtai–Yejituo fault coincide closely with anomaly boundaries: the northern side of Ninghe–Changli fault exhibits positive anomalies while the southern side is negative; conversely, the northern side of Fengtai–Yejituo fault shows negative anomalies and the southern side is positive, illustrating the basin-controlling and uplift-controlling roles of these faults. The primary Tangshan fault zone in the epicentral area is marked by a prominent positive gravity anomaly, with boundaries aligning with the strikes of secondary faults in a continuous banded configuration. This feature corresponds to an intermittent distribution of high-density bodies in the upper crust, reflecting tectonic differentiation between the North China Plain and the Yanshan Fold Belt. These high-density bodies are interpreted as the cooling products of deep-seated magmatic upwelling. Seismicity exhibits a pronounced gradient-constrained characteristic, clustering within the RGAs gradient zones at the interface between high-density bodies and the surrounding low-density media, while also aligning closely with the strike of the Tangshan fault. This indicates that under the influence of regional tectonic stress, the abrupt physical property transition surfaces within this depth interval produce significant rheological contrasts and stress locking.

**Figure 6:**
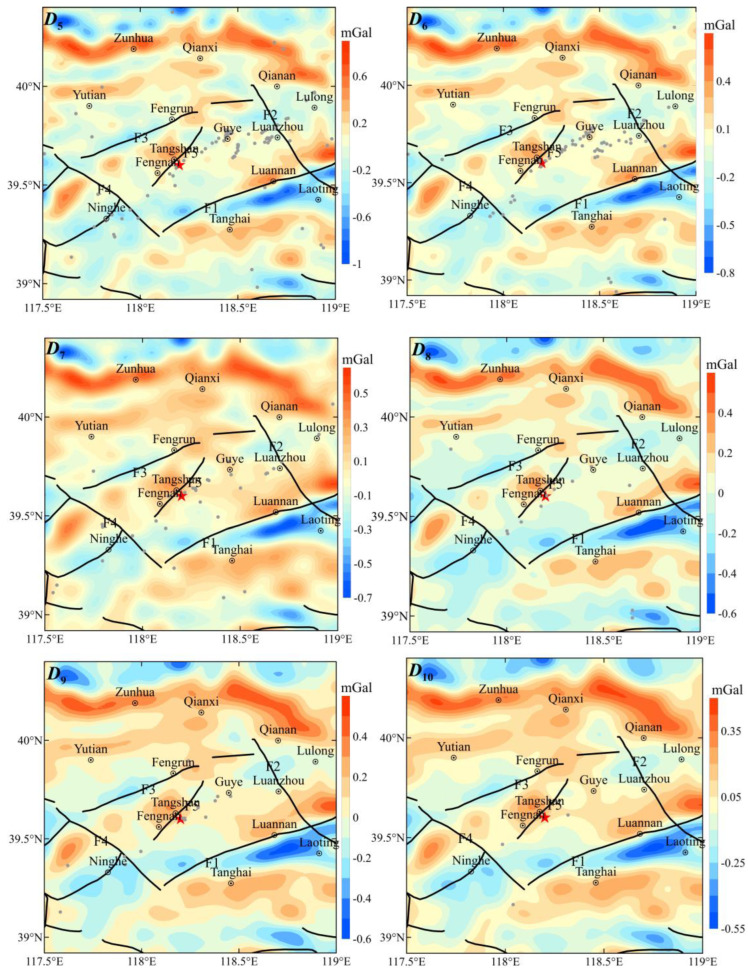
5th-to-10th order wavelet detail components (*D*_5_~*D*_10_) of the RGAs in the Tangshan seismic zone. The red star indicates the epicenter of the 1976 Tangshan Ms7.8 earthquake.

Figure 6 displays the 5th to 10th order wavelet detail components (*D*_5_~*D*_10_) of the RGAs within the study area. The 5th and 6th order wavelet details primarily reflect RGAs at mid-crustal depths of approximately 15 km. At these levels, the positive anomaly within the northern Zunhua–Qianxi–Qian’an region contracts, exhibiting a more concentrated morphology. While the spatial wavelengths of the positive and negative anomalies expand, their magnitudes gradually attenuate. The 7th to 10th order wavelet details, corresponding to depths of approximately 18 km to 26.45 km, reflect the material distribution within the lower crust. In these components, the range of positive and negative anomalies shrinks further, yielding an overall “high in the north and low in the south” distribution pattern. This reflects the crustal thinning trend during the transition from the northern Yanshan Uplift to the North China Plain basin in the south. A localized high-value positive anomaly persists near Tangshan; however, as depth increases, the extent of this high-value anomaly gradually contracts, and its peripheral detail features vanish. This suggests that the positive gravity anomaly in the Tangshan seismic zone extends into the lower crust, reflecting the state of mass migration during the activity of the Tangshan fault. The positive gravity anomaly associated with the Tangshan fault zone merges with the high-density anomalies of the Archean crystalline basement in Zunhua–Qianxi, forming an uninterrupted, continuous NE-trending belt. These high-density geologic bodies are segmented by faults and distributed in an alternating arrangement, which closely corresponds to the regional geomorphology characterized by alternating uplifts and depressions [41].

### 3.2. Vertical Characteristics of Wavelet Details

**Figure 7:**
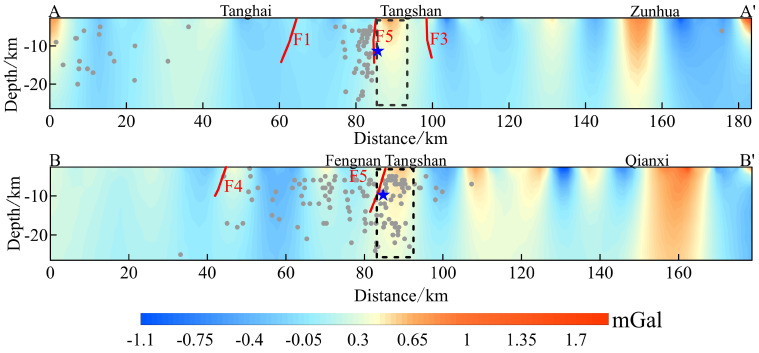
Vertical cross-section of wavelet details across the Tangshan seismic zone. AA’ and BB’ represent extracted gravity profiles crossing the epicentral core and primary faults (as shown in Figure 3). Red lines denote faults; the gray dots denote the historical seismicity data; the blue star indicates the epicenter of the 1976 Tangshan Ms7.8 earthquake. The black dotted box denotes the positive anomaly region corresponding to the epicenter of the Tangshan earthquake.

To better analyze the relationship between faults and RGAs and to reveal the constraints imposed by deep crustal heterogeneities on seismic activity, two gravity profiles were extracted across the study area (see Figure 3), with the results shown in Figure 7. Overall, the crustal RGAs within the seismic zone exhibit a significant spatial evolution from shallow dispersion to deep aggregation with increasing depth: shallow anomalies are mostly distributed as scattered, narrow bands, while deep RGAs gradually coalesce. Profile AA’ intersects the Ninghe–Changli fault, Fengtai–Yejituo fault, and Tangshan fault perpendicularly and is characterized by a “narrow and deep” structural architecture. The earthquake sequence is distributed in a steep, linear fashion along the primary Tangshan fault, corresponding to a notably sharpened RGAs gradient. This indicates that tectonic stress in this direction is highly concentrated within a single primary rupture surface and the extremely narrow physical property transition zones flanking it. Consequently, it is inferred that the primary Tangshan fault is likely a high-angle, deep-seated structure penetrating the middle and lower crust, potentially offsetting the Moho and dominating vertical stress accumulation and energy release. Profile BB’ extends essentially along the strike of the Tangshan fault, exhibiting a broad and complex lateral heterogeneity, which reflects that seismic activity is distributed in close spatial proximity to the Tangshan fault zone. Within the Tangshan segment, the wavelet detail signals are dominated by localized positive anomalies; although amplitudes attenuate with increasing depth, the positive anomalies persist to at least 26 km, revealing the presence of deep-seated high-density roots. Furthermore, fault intersections generally coincide with steep changes in gravity gradients or localized fluctuations. The amplitude of these disturbances is significantly higher in the shallow crust than at depth. This confirms the role of the faults as density boundaries and reflects a tectonic evolutionary transition from pronounced shallow anisotropy toward relative homogeneity at greater depths.

## 4. Discussion

### 4.1. Deep Dynamics of the Tangshan Seismic Zone and Its Link to Craton Destruction

As a typical intraplate strong earthquake event within the destruction zone of the NCC, the Tangshan earthquake is characterized by a highly complex tectonic background. The region is situated on the eastern flank of the Taiyuan–Yanqing BGAs gradient zone (the central segment of the Great Gravity Gradient Zone in Eastern China) and the Moho depth mutation zone. The epicentral area occupies a geodynamic transition between the Yanshan Uplift and the North China Basin. Driven by the big mantle wedge mechanism induced by the subduction and subsequent rollback of the Western Pacific Plate, a typical “deep dynamic forcing—shallow structural response” coupling system has been established [42].

**Figure 8:**
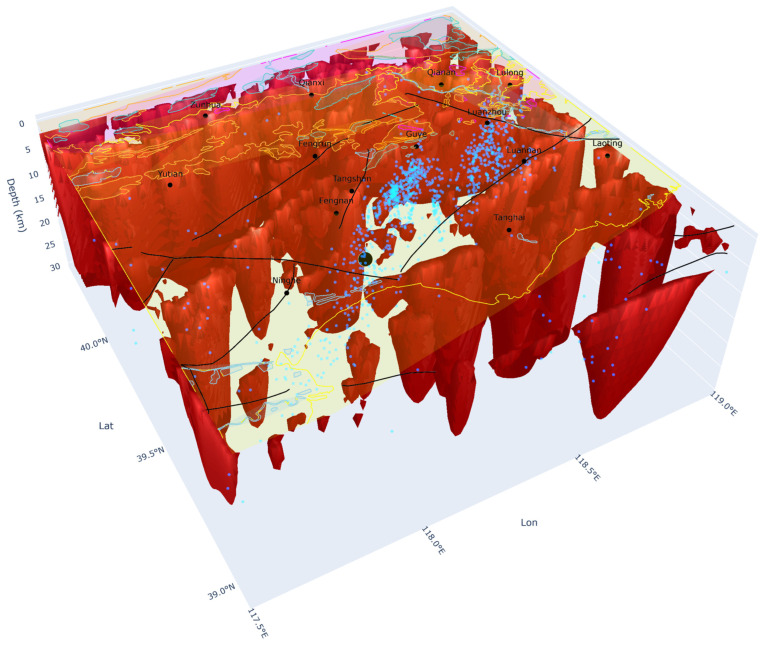
3D structural and hypocenter distribution model of the Tangshan seismic zone. Red isosurfaces represent positive gravity anomalies (high-density bodies), revealing the distribution of deep-seated material. The black circle denotes the epicenter of the Tangshan Ms7.8 earthquake, while blue dots indicate the spatial distribution of the earthquake sequence. Black lines represent the 3D framework of the major fault zones. The pink, green and blue lines represent the stratigraphic boundaries in Figure 1.

Deep seismic reflection profiling confirms that the crustal thickness in the seismic zone ranges from 32 to 34 km, with the Moho depth increasing progressively from east to west [12,13]. The Tangshan deep fault, the primary seismogenic structure, exhibits a characteristic “flower structure” in the shallow crust and penetrates the crust–mantle transition, resulting in a distinct Moho offset across the fault [17]. In this study, the vertical profiles of wavelet details and the 3D structural and hypocenter distribution model of the Tangshan seismic zone (Figure 8) clearly illustrate the “convergence” characteristics of RGAs at depth. Within the 15–26 km depth range, positive gravity anomalies in the Tangshan segment exhibit continuous, columnar, or banded morphologies, demonstrating robust vertical connectivity without significant physical property offsets or structural barriers. This deep-seated connectivity is highly consistent with the deep high-conductivity anomalies reported by Cai et al. [5], and the thermal upwelling signatures identified by Li et al. [43]. These integrated geophysical observations collectively suggest that mantle material undergoes upward underplating or emplacement along deep-seated faults, providing a critical conduit for the upward transmission of energy.

### 4.2. Multi-Scale Evolution of Gravity Anomalies and Its Deep Convergence–Shallow Dispersion Structure

The multi-scale wavelet separation results (Figure 5, Figure 6 and Figure 7), combined with the 3D structural map of the Tangshan seismic zone (Figure 8), clearly illustrate the spatial evolution of RGAs with increasing depth. The “Shallow Dispersion” characteristics exhibited by the 1st-to-4th order detail signals (depth < 8 km), along with the surface model contours, reveal that the epicentral area is defined by the Tangshan Rhombic Fault Block. This configuration is bounded by NE-trending (Ninghe–Changli fault, Fengtai–Yejituo fault) and NW-trending (Luanxian–Laoting fault, Jiyunhe fault) faults, reflecting shallow-crustal fragmentation driven by stress dispersion and tectonic adjustment.

With increasing depth, the 8th-to-10th order wavelet details (corresponding to 21 km–26.45 km) exhibit prominent “Deep Convergence” characteristics with enhanced interconnectivity between gravity highs. Laterally, the anomaly field is significantly influenced by the fault-related truncation, manifesting as an alternating distribution of positive and negative gravity anomalies along both sides of the NE-trending faults (Ninghe–Changli fault, Fengtai–Yejituo fault, and Tangshan fault). High-value anomalies along the Ninghe–Fengnan–Tangshan line are distributed in an intermittent, beaded pattern, reflecting intense lateral heterogeneity within the crust. Vertically, previously isolated anomalies begin to converge at depth. In the Qianxi–Qian’an–Lulong region and the Tangshan–Guye–Luanxian core area, high-value anomalies expand and interconnect. This evolution—from narrow, shallow bands to large-scale, deep-seated high-value zones—reveals coupled Deep–Shallow geodynamic framework. These deep-seated mass aggregations corroborate material accumulation driven by asthenospheric upwelling, while the shallow banded anomalies correspond to sharp physical property transitions between high-density intrusive bodies and low-density host rocks. This fundamental contrast provides the structural basis for the gradient-localized distribution characteristic of major earthquakes [44,45,46].

### 4.3. Seismogenic Mechanism and Stress Accumulation of the Tangshan Earthquake

Integrating the characteristics of RGAs at various source depths with hypocenter, the seismogenesis mechanism of the Tangshan earthquake follows a process of “Deep Dynamic Loading—Shallow Locking and Energy Storage—Instability, Rupture, and Energy Release.” The fundamental driving force stems from the coupling between mantle magmatic activity and fault structures. Lithospheric delamination in the eastern NCC triggered the partial melting of the upper mantle; subsequently, mantle-derived magma underwent upward underplating or emplacement along the Tangshan deep fault, promoting the progressive expansion of pre-existing fracture surfaces within the deep fault zone. The connectivity of deep gravity highs revealed by the 5th-to-10th order wavelet details (*D*_5_~*D*_10_), together with the vertical distribution of high-value anomalies along the Tangshan segment, corroborates this upward magmatic migration. Furthermore, the asthenospheric upwelling induced by the subduction and rollback of the Pacific Plate exacerbated Moho uplifting and lateral compression, providing a sustained dynamic source for shallow tectonic activities [47,48].

The shallow, banded positive gravity anomalies identified in the 1st-to-4th order wavelet details suggest that the lithified sedimentary sequence has impeded the upward migration of magma. Consequently, sharp gravity gradients have developed at the interfaces between high-density intrusive bodies and low-density host rocks. Under the persistent influence of the regional NW-SE trending principal compressive stress, these zones are highly susceptible to becoming locked regions characterized by significant stress and strain concentration. Simultaneously, the high-angle (70°~80°), west-dipping, oblique-thrust–strike–slip structure of the shallow Tangshan fault further increases the normal stress on the fault plane. This strengthens the mechanical locking of the crustal media and facilitates the continuous accumulation of strain energy within the 10~25 km deep seismogenic layer [19].

As illustrated in Figure 8, hypocenters are densely clustered along the margins of high-value gravity anomalies and at fault intersections, particularly within the 10~25 km seismogenic layer. Once the stress accumulation within the locked zone reaches a critical failure threshold, the shallow faults undergo rapid oblique–thrust–strike–slip displacement, triggering a sudden release of accumulated energy [20,49]. The widening of gravity anomaly bands along the fault strike, as revealed by the 1st-to-4th order wavelet details, combined with the “narrow-and-deep” structural features identified in Profile AA’, indicates that faulting processes drive the development of shallow flower structures while simultaneously producing significant offsets within the middle-to-lower crust and the crust–mantle transition zone. This structural configuration facilitates a synchronous release of tectonic energy across both shallow and deep-seated crustal levels.

The Tangshan earthquake and the 1679 Sanhe–Pinggu Ms8.0 earthquake share remarkable similarities in their tectonic mechanisms, collectively corroborating a unified geodynamic model for strong earthquakes within the destruction zone of the NCC [30,41]. Both events are situated at the intersection of gravity anomaly gradients and abrupt Moho transitions, underpinned by a seismogenic mechanism driven by “magma intrusion–fault coupling”. However, multi-scale gravity analysis reveals that shallow RGAs in the Tangshan seismic zone are more diffuse, accompanied by a more pronounced sharpening of the gradient zones. This reflects a thicker shallow sedimentary cover and stronger lockingof high-angle faults in the Tangshan region. Consequently, this area is characterized by more extensively developed shallow flower structures, longer-term stress accumulation, and a tighter clustering of hypocentral depths (10~25 km)—predominantly concentrated along the transition zones between high-and-low density media within the middle-to-lower crust.

## 5. Conclusions

Through the application of multi-scale wavelet decomposition to RGAs in the Tangshan seismic zone, this study systematically elucidates the coupling characteristics between deep and shallow crustal structures, as well as their controlling influence on the seismogenesis of major earthquakes. The principal conclusions are as follows:

1. The RGAs within the Tangshan seismic zone exhibit a pronounced spatial evolution characterized by “Shallow Dispersion and Deep Convergence.” The 1st-to-4th order wavelet details (depth < 10 km) reflect the fragmented architecture of the “Tangshan Rhombic Fault Block” and its shallow flower structures, which are crossed thoroughly by multiple fault sets. In contrast, the 5th-to-10th order details (13.16 km ~26.45 km) reveal a deep-seated trend where anomalies coalesce into continuous banded formations. This deep convergence reflects a regional geodynamic framework governed by the upwelling of deep, mantle-derived thermal material.
2. The 3D structural model and wavelet detail profiles of the Tangshan seismic zone demonstrate that the primary seismogenic Tangshan Fault manifests as a prominent zone of sharp gravity gradients. Furthermore, Profile AA’ reveals that tectonic stress is heavily concentrated within these vertically narrow gradient zones. Functioning as a high-angle, deep-seated structure, the Tangshan fault plays a dominant role in the vertical transmission of mantle-driven dynamics and the focusing of strain energy into the upper crust.
3. The Tangshan earthquake is fundamentally driven by Big Mantle Wedge dynamics induced by the subduction and rollback of the Western Pacific Plate, which fuel the sustained upwelling of deep thermal material. Within the middle crust (10–25 km), the significant rheological contrast between the rigid Archean basement and the Cenozoic sedimentary strata creates a highly efficient locking and energy-storage zone. Ultimately, the progressive accumulation of tectonic stress culminates in mechanical instability and catastrophic rupture along the high-angle fault systems in the upper crust.

## Data Availability

The seismic event catalog is available through CENC (http://www.ceic.ac.cn/) and accessed on 1 January 2026. All geological data supporting the findings of this research are publicly available through the Geocloud platform “https://geocloud.cgs.gov.cn/ (accessed on 1 January 2026)”, which is hosted by the China Geological Survey and provides comprehensive geological data resources.
